# Evidence of tsunami forces shaping clifftop boulder fields from integrated morphometric and numerical modeling approaches

**DOI:** 10.1038/s41598-025-24741-x

**Published:** 2025-11-20

**Authors:** Àngels Fernández-Mora, Bernadì Gelabert, Lluìs Gómez-Pujol, Francesc X. Roig-Munar

**Affiliations:** 1 Earth Science Research Group, Department of Biology, University of the Balearic Islands, Palma, Spain; 2 Balearic Islands Coastal Observing and Forecasting System, Palma, Spain; 3 Palma, Spain

**Keywords:** Cliff-top deposits, Tsunami, Modelling, Natural hazards, Geology

## Abstract

Cliff-top boulder deposits are rare but striking geomorphic features found along high-energy coastlines. Their formation mechanisms—whether from storm waves or tsunamis–remain a subject of scientific debate. This study examines tsunami-induced boulder transport on the steep southern coast of Mallorca Island (Western Mediterranean), integrating detailed morphometric analysis with high-resolution simulations using a non-linear numerical model. Boulder characteristics, including volume, elevation, distance from cliff edges, and orientation, were mapped and used to compute mobilization thresholds under subaerial, submerged, and joint-bounded block (JBB) conditions. Our simulations tested 360 tsunami scenarios varying in wave height, period, and direction. Results show that wave height alone is insufficient to explain boulder transport; instead, velocity thresholds–particularly for saltation and JBB lifting—provide more accurate indicators. The model successfully replicates tsunami propagation, cliff impact, and inland flooding, revealing that only long-period, high-magnitude waves from southern directions exceed the thresholds needed to displace the observed boulders. Boulder deposits differ across the study area: smaller, higher-elevation blocks in the western sector are only mobilized by a narrow range of extreme tsunami conditions, while larger, lower-elevation eastern boulders respond to a broader spectrum. Storm wave simulations failed to reach or displace the boulders, strengthening the tsunami hypothesis. These findings highlight the critical role of numerical modeling in tsunami hazard assessment and call for re-evaluation of other cliff-top deposits globally. The methodology presented here demonstrates a robust, multidisciplinary approach to interpreting boulder emplacement and contributes to refining coastal hazard mapping in tsunami-prone regions.

## Introduction

Tsunamis are long gravity waves that are generated in the ocean by abrupt geophysical events such as undersea earthquakes, submarine landslides, or volcanic eruptions. Among them, those caused by undersea earthquakes are most frequent^1^. Sometimes these processes work in conjunction with each other to enhance the amplitudes of the tsunamis. The energy carried by tsunami waves can cause significantly greater damage than typical storm surge waves due to their longer wavelengths, higher velocities, and ability to propagate across entire ocean basins with minimal energy loss. The primary effects of tsunamis in coastal areas include extensive flooding, high-energy wave impacts, coastal erosion, strong currents, and their ability to transport large volumes of debris.

In recent decades, significant advancements in monitoring systems, data acquisition technologies, and numerical modeling techniques have facilitated precise recording, analysis, and investigation of the complex processes governing tsunami generation, propagation, and subsequent inundation impacts. These developments have been particularly evident in recent major events such as the 2004 Indian Ocean tsunami^2,3^, the 2016 Fukushima tsunami^4,5^, and the recent 2022 Hunga Tonga-Hunga Ha’apai eruption^6,7^. The high-resolution data obtained from these events have substantially improved the accuracy of tsunami hazard quantification and risk assessment. However, such comprehensive datasets remain largely confined to these recent occurrences.

Although there are no data sources comparable to those available for recent events, tsunamis have left behind geological evidence in the form of tsunamites, sedimentary deposits that provide invaluable insights into past seismic activity and its impacts on coastal regions. These deposits are typically characterized by unique sediment layers that can include a mix of marine and terrestrial materials, anomalous boulder placements, and microfossils that indicate sudden marine incursion onto land. Studies of tsunamites around the world, from the Mediterranean to the Pacific rims, have enabled scientists to reconstruct the frequency, magnitude, and effects of historical tsunamis, extending our understanding of these phenomena beyond the limits of written records. Ultimately, the analysis of tsunamites not only helps in the historical understanding of tsunamis but also aids in refining current models used for predicting potential future tsunami risks.

The formation of cliff-top boulder deposits is a subject of ongoing debate among geologists and oceanographers, primarily concerning the differentiation between deposits left by tsunamis and those created by other high-energy events like storms. Critics argue that certain geological features attributed to tsunamis deposits, such as the size, the sorting of sediments or the imbrication of larger clasts, can similarly result from storm surges, making it challenging to definitively attribute such layers to tsunami activity without additional contextual evidence. Focusing on tsunami boulder deposits, large clasts displaced from coastal or nearshore environments by high-energy tsunami waves, it is evident that their size, orientation, and spatial distribution are influenced by hydrodynamic forces, substrate characteristics, and coastal geomorphology. This type of deposit is found worldwide (^8–11^, among others).^12^, along with subsequent studies by^13,14^, developed parametric approaches to elucidate the formation mechanisms of tsunami boulder deposits. These approaches are based on boulder morphometrics and provide wave height and velocity thresholds for boulder mobilization, considering various mobilization conditions, including submerged, subaerial, and boulder lifting scenarios. These parameterizations enable the estimation of the energy required for the transport of boulders. Several studies focused on the identification and genesis of these deposits rely on these formulations to ascertain their origins (^15–19^, among others).

However, the processes of wave propagation, both for tsunamis and storm waves, are highly nonlinear and complex, particularly as they approach shallow waters^20^. Therefore, parametric or simplified approaches to these processes are insufficient for determining the specific phenomena that have caused the displacement of boulders at the wave-rock impact point. This complexity underscores the necessity for a multidisciplinary approach that integrates geomorphological data with historical tsunami records and contemporary modeling to accurately identify and attribute the origins of cliff-top boulders associated with tsunamis.

In this study, we investigate the potential mechanisms underlying the formation of cliff-top boulder deposits by integrating detailed geomorphometric measurements with numerical modeling, specifically focusing on boulder deposits along the southern coast of Mallorca Island (Balearic Islands, Western Mediterranean).

**Fig. 1:**
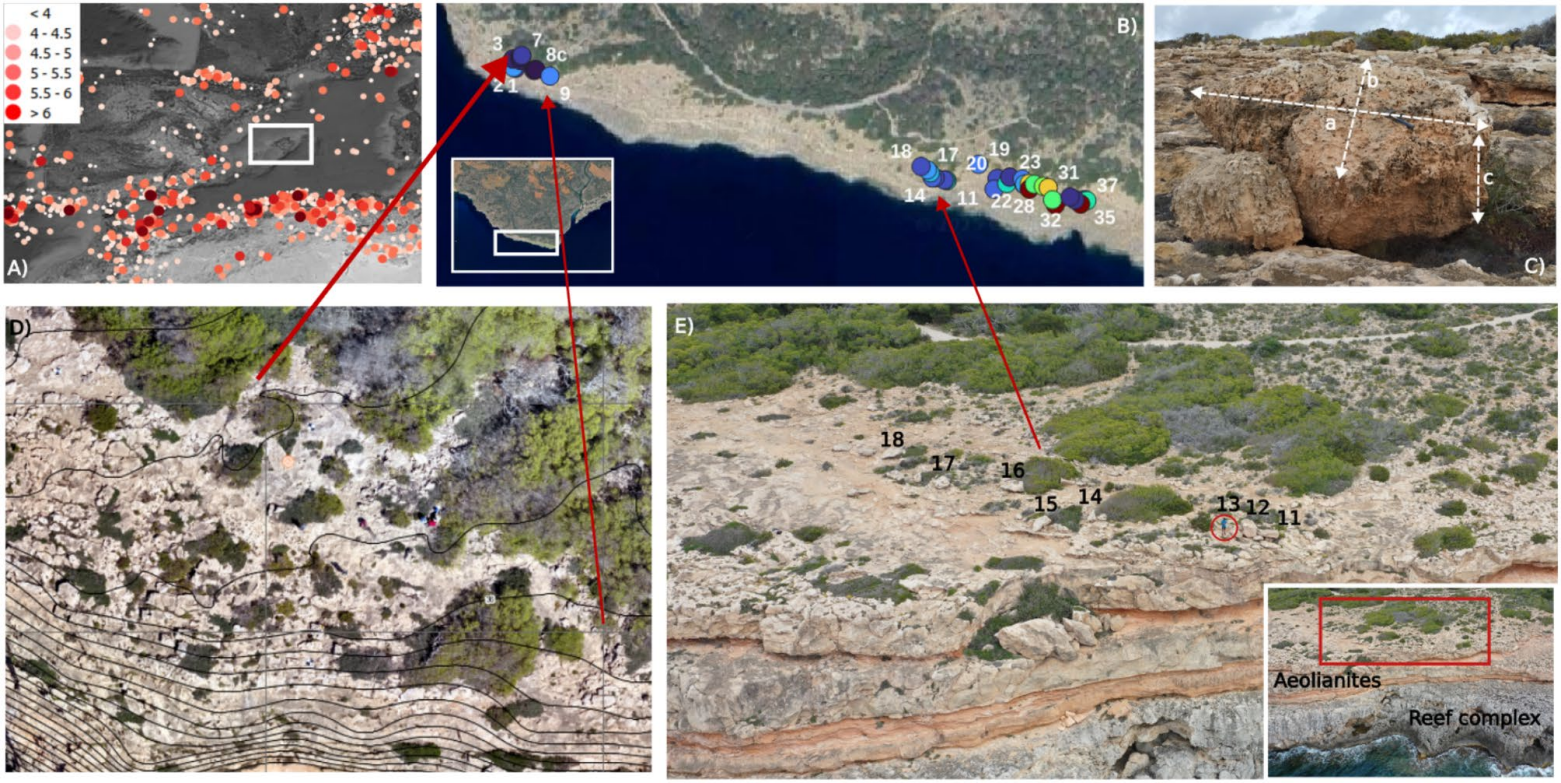
(**A**) Location of the Balearic Islands (Minorca, Mallorca, Ibiza) and USGS earthquake data since 1700 at the Western Mediterranean (through Qquake QGIS software,^21^). (**B**) Boulders location at the study site. Colours stand for boulder volume. (**C**) Boulders axes definition. (**D**) Western boulder field at Es Bancals. Boulders are located at 37 m above sea level. (**E**) Eastern boulder field at Es Bancals. Numbers refer to boulders. A person in a circle as scale.

## Es Bancals cliff-top boulders

A flat lying Upper Miocene Reef complex builds up Es Bancals study site (location on Fig. 1A,B), in the south of Mallorca Island (Spain). On top of the Reef Complex, Middle Pleistocene carbonate aeolianites and colluvial deposits exist^22^. A single cliff, 34 m high, formed by the two units, exists at the western sector (Fig. 1C). To the best of the authors’ knowledge, this sector hosts the highest-positioned coastal boulders reported in the Western Mediterranean: 37 m above sea level and at a 34 m distance from the cliff edge, with volumes of up to 0.99 m$^3$ (Fig. 1D). A wave-cut platform at 10 m a.s.l. separates both units in the eastern sector of the study area (Fig. 1E). In the Eastern Sector the three eolianites levels are more resistant than the colluvial (mostly marls) deposits and form two minor (3–4 m high) topographic steps, separated by two flat areas located at 15–18 and 22 m above sea level. At these upper terraces, large imbricate coastal boulders appear (Fig. 1E). Further details of boulder position and size are provided at^23^.

The southern part of Mallorca Island, where Es Bancals study site is located, is the continental area closest to the main tsunamigenic sources of the Western Mediterranean which, according to^24^, are located offshore Algeria (Fig. 1A). At least six tsunamis have been catalogued over the last centuries affecting the Balearic Islands: in 1365, 1756, 1802, 1856, 1980 and 2003^25^.^26^ described in his chronicles a very large tsunami in 1756 that flooded more than 2.4 km inland in Cala Santanyì (SE Mallorca).

The most recent tsunami reaching the Balearic Islands shores took place on May 21, 2003 and was generated by the Zemmouri earthquake (offshore Algeria). This tsunami affected parts of Spain, France and Italy, but it mostly damaged harbour facilities south and east of the main Balearic Islands, including 3 m high waves on Ibiza Island. Tsunami simulations of the 1856^27^ and 2003^28^ events predicted tsunami wave impact in the exact places where cliff-top boulders on the Balearic coasts appear (Fig. 1 B). Despite storm wave height and energy being much larger from the north and west at the Balearic Islands^29^, these boulders are located in the eastern and southern areas of the islands^18^.

## Morphometric analysis and mobilization thresholds

Cliff-top boulders at the study area are primarily accumulated in two distinct groups: the western sector (WS) and the eastern sector (ES). The WS includes 11 studied boulders located at an average height of $36.46\pm 1.22\,\hbox {m}$ above sea level and $37.14\pm 6.88\,\hbox {m}$ from the cliff edge. These boulders exhibit an orientation of $220\pm 39.94^{\circ }$ relative to the north. The boulders are relatively homogeneous, with average dimensions of $1.08\times 0.70\times 0.34\,\hbox {m}$ and an average volume of $0.31\pm 0.31\,\hbox {m}^{3}$. The ES contains 29 studied boulders at an average height of $20.24\pm 3.45\,\hbox {m}$ and $49.09\pm 16.11\,\hbox {m}$. In contrast to the WS, the boulder dimensions in the ES are more heterogeneous, with volumes ranging from 0.23 to 6.72 m³. Notably, they are spatially distributed from west to east within the sector, showing a gradual increase in volume along the East cliff ledge (Fig. 1 C). Their average orientation aligns with that of the WS at $220.53\pm 22.81^{\circ }$, with a dip angle towards the sea of $26.95\pm 13.73^{\circ }$. Boulders characteristics for both sectors are detailed in the Table 1 and Table 2, respectively.

**Fig. 2:**
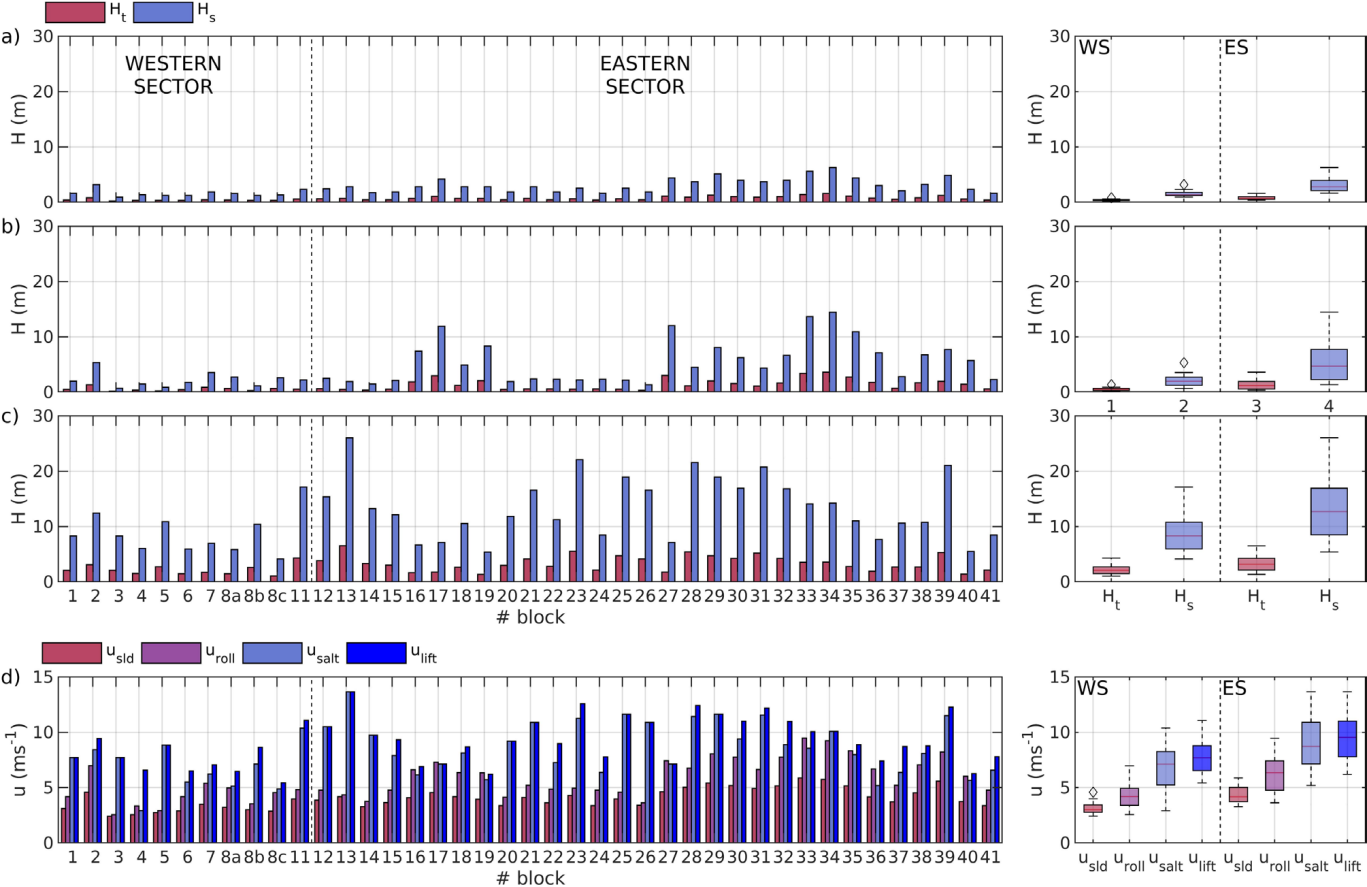
Tsunami (red) and wind wave (blue) $H_t$ and $H_s$ mobilization threshold for each block considering (**A**) subaerial, (**B**) submerged and (**C**) JBB conditions. Right panels show the boxplot of $H_s$ and $H_t$ for both sectors. In (**D**) Velocity thresholds for each block considering sliding, rolling, saltation and JBB lifting conditions. Vertical dashed line separates western and eastern sectors. Right panels show the boxplot of $u_{slid}$, $u_{roll}$, $u_{salt}$ and $u_{lift}$ for both sectors.

Three initial conditions are considered to determine the boulders mobilization threshold: (1) subaerial boulders, which are fully exposed above mean sea level (MSL); (2) submerged boulders, which remain consistently underwater; and (3) Joint-Bounded Boulders (JBB), whose size and shape are primarily controlled by pre-existing joints, fractures, or planes of weakness in the bedrock, rather than by surface processes such as abrasion or weathering. JBBs are typically located at or near the transition zone between the subaerial and submerged environments. The modified^12^ equations, detailed in Table 3, are applied to each boulder in terms of tsunami wave height $H_t$ and sea wave heights $H_s$ and of velocity *u*. These thresholds provide the wave height at the impact point able to mobilize the boulder. Figure 2A–C show the resulting values of $H_t$ and $H_s$ for each condition, along with boxplots for each threshold heights and sector. Results are also summarized in Tables 4 and 5. Notable differences between the western sector (WS) and the eastern sector (ES) are evident for both subaerial and submerged conditions, with the WS consistently exhibiting lower mobilization thresholds for both $H_t$ and $H_s$ across all conditions.

For subaerial conditions, the mean $H_t$ threshold values are 0.40±0.16 m for the WS and 0.79±0.31 m for the ES, while the $H_s$ thresholds are 1.62±0.64 m and 3.15±1.25 m , respectively. In submerged conditions, tsunami and wave height thresholds increase for both sectors, with $H_t$ values of 0.55±0.64 m (WS) and 1.87±1.01 m (ES) and $H_s$ values of 2.20±1.33 m (WS) and 7.49±4.02 m (ES). Under JBB conditions, both tsunami and wave height thresholds increase for both sectors (WS: 2.19±0.93 m and 8.75±3.74 m; ES: 3.39±1.39 m and 13.58±5.56 m), remaining consistent the trends between sectors. These variations can be directly attributed to differences in boulder dimensions between the two sectors.

Regarding velocity thresholds for boulder mobilization type (Fig. 2d) in subaerial/submerged conditions, as expected, the sliding velocity threshold ($u_{sl}$) is smaller than the rolling ($u_{rol}$) and saltation ($u_{slt}$) thresholds. For the WS, the thresholds range from 3.17±0.64 $\mathrm {ms^{-1}}$ for sliding, 4.31±1.25 $\mathrm {ms^{-1}}$ for rolling, and 6.80±2.12 $\mathrm {ms^{-1}}$ for saltation. In the ES, the respective values are 4.33±0.77 $\mathrm {ms^{-1}}$, 6.19±1.63 $\mathrm {ms^{-1}}$ and 8.88±2.26 $\mathrm {ms^{-1}}$. Similar to wave height thresholds, velocity thresholds follow a comparable pattern across mobilization types, with lower velocities observed in the western sector (WS) compared to the eastern sector (ES). The only exception is for saltation, where velocities are relatively consistent across both sectors, although slightly higher in the ES. As expected, JBB velocity thresholds, which represent the saltation/lifting mechanisms of mobilization, are higher in both sectors. These thresholds are 7.77±1.62 $\mathrm {ms^{-1}}$ for the western sector (WS) and 9.40±1.92 $\mathrm {ms^{-1}}$ for the eastern sector (ES).

## Modelling tsunami waves and boulder transport capacity

Morphometric analysis and analytical equations provide the tsunami and wind waves thresholds in terms of height and velocity at the initial position of the blocks to be mobilized. However, wave propagation processes are nonlinear and complex, involving various mechanisms such as shoaling, breaking, and energy dissipation. Therefore, determining which tsunami wave conditions may impact the cliff edge with sufficient height and velocity requires the use of advanced modeling tools.

**Fig. 3:**
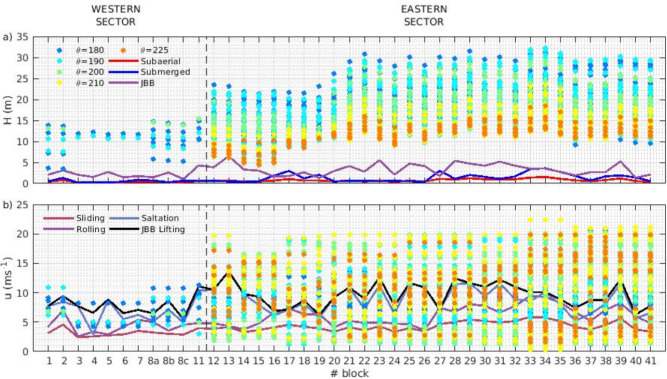
Maximum wave height *H* (**A**) and total velocity *u* (**B**) at the impact point of each boulder for all the tsunami waves simulations. Results are grouped in colours by offshore direction $\theta _0$, the brighter the colour the higher the period *T*. Background lines stand for wave height (**A**) and velocity (**B**) thresholds. Total velocity magnitude *u* is computed considering the $u_x$ and $u_y$ components of the velocity field.

To this end, and to assess the transport capacity of cliff-top boulders, the propagation of different tsunami waves, with varying heights, periods, and directions, has been simulated from offshore depths towards the cliff using a Fully Nonlinear Boussinesq Wave model^30^, totaling 360 simulations of tsunami waves. Further details of the numerical model as well as model setup and assumptions can be found in Section Methods.

For all simulations, the point of contact between the incoming wave and the position of each boulder is extracted in terms of *H*, the $u_x$ and $u_y$ components, and the corresponding velocity magnitude *u* is calculated. These variables have been compared with the wave height and velocity thresholds provided in the previous section.

Figure 3 presents the maximum water column height (*H*) and the total flow velocity (*u*) at the boulder impact location, as obtained from the numerical simulations. Each point along the vertical axis corresponds to one of the initial tsunami wave conditions tested in the numerical simulations. Note that if the water does not reach the impact position, no result is shown. The results are color-coded by approximation angles, with color intensity representing the wave period. In 54.4% of the simulations, the water never reached the clifftop at any boulder position. Of the simulations reaching the cliff-top simulations, 100% reach ES and the 4% reach the WS. Regarding the direction of incoming waves reaching the cliff-top, 12.6% correspond to southern incoming waves, progressively increasing to 34.75% for waves from the southwest (SW) direction. Notably, all incoming waves reaching the western sector (WS) originate from the south (S) and south-southwest (SSW) directions. In contrast, wave directions reaching the eastern sector (ES) range from S to SW.

Regardless of the case, all simulations where water reached the clifftop surpassed the three wave height thresholds considered for each boulder (Fig. 3A). However, when analyzing the results in terms of total velocity at each boulder position, it is evident that, unlike the wave height thresholds, not all simulations exceeded the mobilization velocity threshold (Fig. 3B). In the eastern sector, among all simulations where an impact has occurred, 70% exceed the sliding threshold, 60% surpass the rolling threshold, 47% exceed the saltation threshold, and 42% surpass the JBB lifting threshold. In the western sector, among the simulations that result in an impact, 99% exceed the sliding threshold, 86% surpass the rolling threshold, 47% exceed the saltation threshold, and 32% surpass the JBB lifting threshold.

**Fig. 4:**
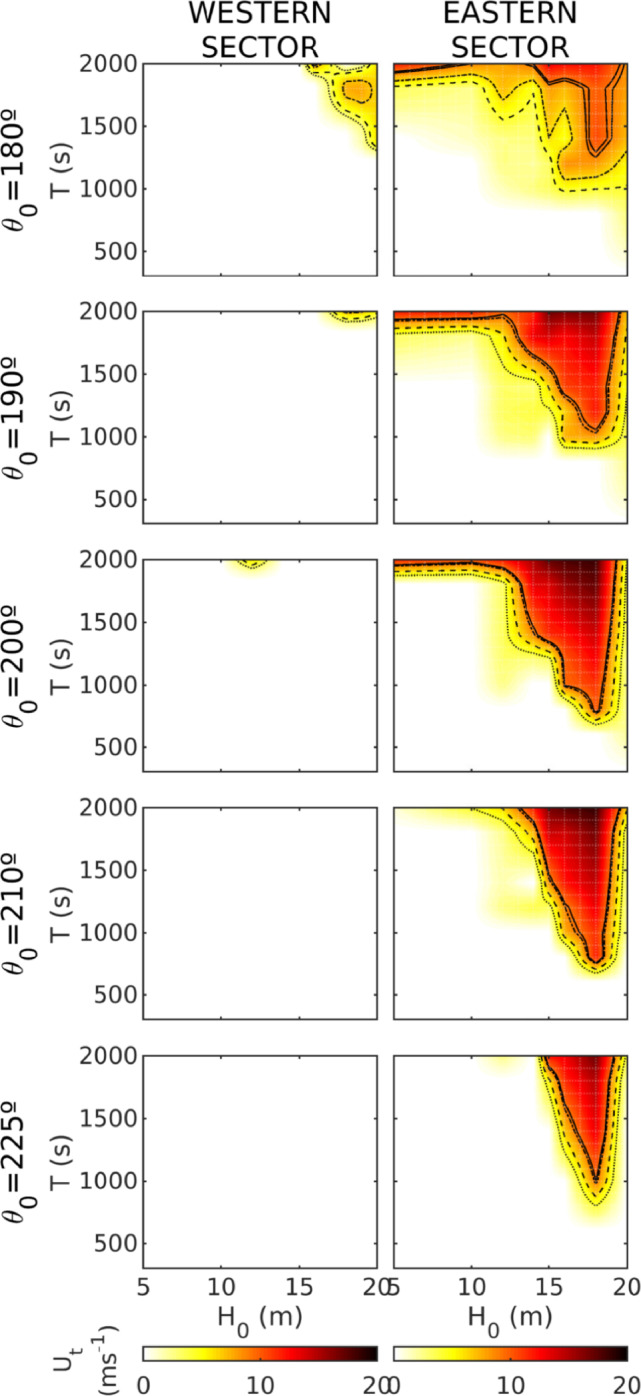
Mean velocity at the boulders position at the impact instant respect to all the offshore wave heights and periods and directions for both sectors. Black lines stand for: sliding velocity threshold, point lines; rolling, dashed lines; saltation, dot-dashed line; JBB saltation/lifting, solid line.

To ease the interpretation and analysis of model results, the mean velocity per sector, modeled at the position of the boulders for the different values of *H*, *T*, and $\theta$ considered, has been computed (Fig. 4). Contour lines represent the different thresholds associated with mobilization types (sliding, rotation, saltation and JBB lifting). Model results indicate that boulder mobilization at the cliff-top in the WS sector is restricted to particularly high and long waves. Under these conditions, the primary transport mechanisms are associated with subaerial and submerged scenarios, most likely through sliding and rolling transport. Notably, such conditions in the WS sector are only feasible with southward incoming tsunami waves. In contrast, the ES sector exhibits a broader range of offshore conditions capable of mobilizing boulders, regardless of the specific transport type or wave direction. Notably, incoming wave angles around $200^0$ present the most likely conditions for boulder displacement across all scenarios.

### Formation of Es Bancals boulder deposits

Morphometric thresholds and modelling results point out that tsunami forcing is a plausible mechanism of formation of these deposits. The morphometric analysis reveals that smaller blocks with lower mobilization thresholds are concentrated in the WS at higher elevations, whereas larger blocks, transported further inland, are found in the eastern sector, which sits at a lower elevation. The numerical results for impact cases, considering wave height and velocity fields, align with these lateral variations along Els Bancals. This increased potential for transport is attributed to the lower elevation of the ES sector, which results in higher wave velocities, further facilitating the transport of boulders.

Regarding block mobilization conditions, these are limited to waves with offshore parameters of $H_t>10$ m and $T>800$ s, falling within the tsunami spectrum. Under these conditions, various block mobilization mechanisms can occur across different sectors, ranging from lower-energy processes (sliding) to more energetic ones (JBB lifting). The most likely mobilization mechanism is sliding, consistent with flume experiments on tsunami boulder transport^31^. Notably, a wider range of mobilization mechanisms is plausible in the eastern sector (ES), with probabilities ranging from 70 to 49%, covering both low- and high-energy processes. In contrast, in the western sector (WS), mobilization is primarily restricted to sliding and rolling, with minimal occurrence of saltation or JBB lifting. It is important to highlight the difference in results when considering wave heights as a threshold versus using the velocity field. In this regard, based on the numerical results, it is evident that wave height thresholds must be treated with caution, as they apply to the wave height at the exact location of boulder displacement, rather than offshore wave conditions. In coastal areas, dissipation and breaking processes play a crucial role, often resulting in a significant reduction in wave height. Furthermore, due to the dynamics of wave motion and the unique morphology of the study area, exceeding the wave height threshold at the impact point does not necessarily imply that the velocity thresholds have also been surpassed. The velocity threshold is then the key variable to determine whether the incoming tsunami waves can transport boulders. Numerical modeling provides valuable insights into wave propagation and the associated velocity fields, underscoring the importance of a multidisciplinary approach in understanding the formation of these deposits.

If we consider the tsunami directions most likely responsible for generating the tsunamite deposits at Els Bancals, these align with previous studies on tsunami generation in the southern Western Mediterranean, at the coast of Algeria (^27,32,33^, among others) as well as with observations of tsunamite deposits along the Western Mediterranean coast (^18,19,34,35^, among others). Based on the results, directions within the SSW-S range could potentially generate these deposits in the eastern sector, while the western sector is limited to SSW directions. Wave propagation processes, including refraction and shoaling across the platform, play a crucial role in facilitating boulder transport for this specific tsunami wave direction.

While the current data do not allow us to determine whether the tsunamite formations in both sectors occurred simultaneously or were caused by the same tsunami event, numerical results suggest that S-component events could potentially generate both deposits. This direction aligns with events associated with the North Algerian seismic sources identified by^32^, particularly the S-3 source. However, events with SSW components are also plausible, corresponding to the S-1 and S-2 seismic sources from North Algeria.

Since the initial position of the blocks before mobilization is unknown, verifying the model results at that position is not possible. However, the current position of the boulders is considered a reliable indicator, as wave heights and velocities would have been greater at their original offshore location compared to their final transported position. In addition, although the precise source of the boulders along the cliff cannot be identified due to the absence of clear erosional or depositional features, their origin is considered local. The c-axis dimensions of the boulders are of the same order of magnitude as the calcoarenite layers underlying them, strongly suggesting that these strata are their source. In addition, the presence of well-developed marine terraces and benchs in the area further supports a local origin for the boulders.

### Numerical modelling of tsunami waves in shallow waters

This study focuses more on the processes of tsunami wave propagation rather than tsunami wave generation. While there are numerical studies that address both the origin and propagation of tsunami waves^32,36^, the resolution of those models may overlook critical dissipation and shoaling processes (transition from kinetic to potential energy), which are essential for assessing tsunami impacts in very shallow areas. By forcing the high-resolution model with long waves that cover the tsunami wave spectrum^37^, the aim is to simulate tsunami waves that have already propagated from their source, having experienced significant shoaling effects as they transition from deep waters to the continental shelf.

**Fig. 5:**
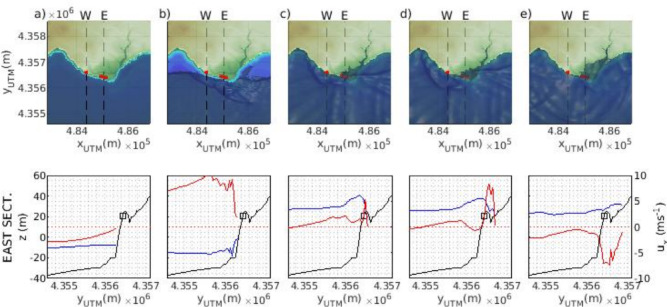
Tsunami waves propagation phases: (**a**) drawback, (**b**) wave approach, (**c**) impact, (**d**) flooding and (**e**) ebb-flood. Upper panels show the tsunami wave bidimensional approach at Els Bancals. Red dots stand for the positions of the boulders. Lower panels show the cross-shore profile of the ES (black line), the sea-surface elevation (blue line, left axis) and the cross-shore velocity component $u_x$ (red line, left axis). Black squares stand for the mean position of the boulders.

Simulations successfully capture the phases of tsunami dynamics in shallow waters. Figure 5 illustrates the different stages of wave propagation for one of the cases considered: drawback flow, approaching wave, impact, flooding and ebb-flood. During the drawback state, sea-surface elevation decreases due to negative (off-shore directed) fluxes in front of the tsunami wave (water retirement functioning similarly to set-down of waves), which results in a lifting of tsunami wave height (Fig. 5A). This process facilitates the transition from vertical to horizontal flow^20^. After this transition, the tsunami wave propagates fast towards the shore, with high onshore directed velocities (Fig. 5B). Wave shoaling results in the amplification of the wave height on approaching the cliff and moving in very shallow waters. Unlike the run-up of a tsunami on beaches or over a constant slope^38,39^, in this case, the tsunami wave climbs the cliff^40,41^ reaching the impact point (boulder position), with significant onshore-directed velocities (Fig. 5C). Once the wave surpasses the cliff-top, it continues propagating inland as a progressive bore^42^, with lower but still considerable velocities (Fig. 5D). Finally, the tsunami retreat, or ebb-flood, occurs with offshore-directed velocities (Fig. 5E). There are numerous observational, experimental, analytical, and numerical studies on tsunami transformation in coastal areas, particularly for constant sloping coasts^43–45^. Special attention has been given to research focusing on tsunami amplification factors caused by shoaling processes (^46–48^, among others). These studies emphasize the importance of tsunami amplification in shallow coastal areas and its significant impact on the total tsunami run-up, which is crucial for accurate tsunami hazard assessment and coastal planning. These works estimate amplification factors ranging from 3 to 6 in regions with gentle slopes. However, tsunami wave amplification due to cliffs has been explored in only a few studies, primarily based on wave tank experiments and numerical modeling of small-scale experiments^40,41^.

Following the definition of amplification factor^40^ as $H=H_m/H_0$ and setting $H_m$ at the impact point, and $H_0$ as the off-shore tsunami wave height, modelling results show a mean amplification factor of $2.28\pm 0.39$ for all the simulations and boulders position. This factor decreases with increasing wave height, ranging from 3.08 to 1.86 from $H_0=10$ to 20 m. This trend is consistent with existing laboratory experiments and numerical studies on tsunami wave interactions with cliffs, which show that smaller incoming waves exhibit higher amplification factors^40,41^. Noteworthy, this consideration should be taken with caution, since these works are based on small-scale experiments with forcing wave heights two orders of magnitude smaller than the current analysis. Clearly, further data is needed to validate these results.

### Tsunami waves or storm waves?

Cliff-top boulders, found in elevated coastal areas, present questions about their transport mechanisms, with both tsunamis and storm waves considered as potential forces^19,43^. Tsunami waves are characterized by long wavelengths and slow, sustained energy over vast distances, while storm waves are shorter, more frequent, and primarily driven by wind, resulting in distinct transport capabilities and coastal impacts.

To assess whether extreme storm wave conditions can reach the velocity thresholds required for different transport mechanisms at the study site, irregular waves corresponding to these extreme conditions are simulated. The offshore storm wave conditions are modeled using a wave spectrum with wave heights ranging from 5 to 10 m, and a peak period of 10 s (minimum frequency $f_{min} = 0.07 \mathrm {s^{-1}}$ and maximum frequency $f_{max} = 0.125 \mathrm {s^{-1}}$), representing the most energetic scenarios in the area (retrieved from Puertos del Estado). To prevent wave dissipation across the computational domain -since tsunami waves are forced in the offshore boundary at 6 km from cliff toe- the storm waves are initiated at a depth of 20 m, approximately 1000 m from the cliff base. Note that $H_s$ threshold from the applied parameterizations to these boulders result in storm wave height values never recorded in 60 years of records.

**Fig. 6:**
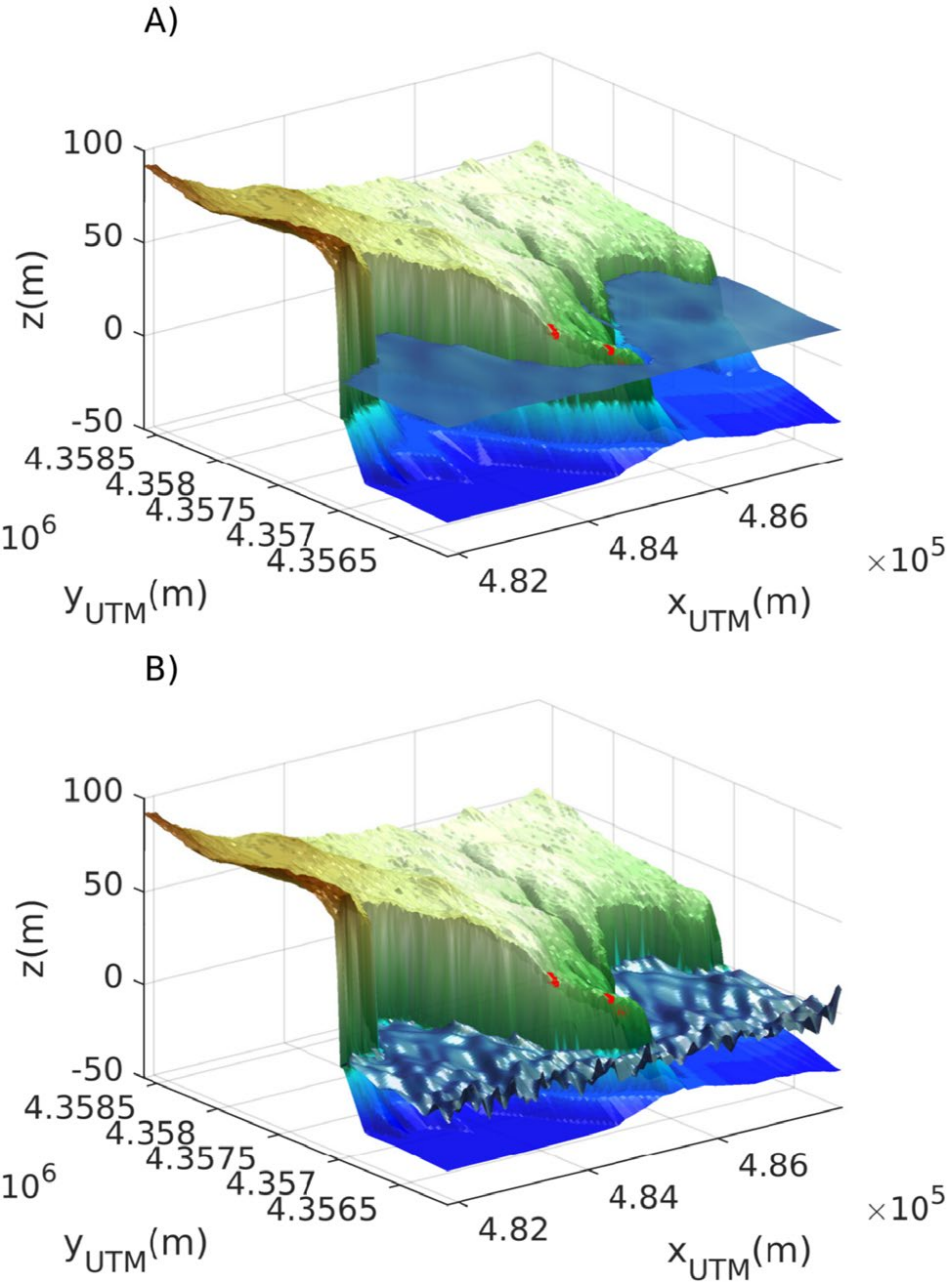
Sea surface elevation at Els Bancals on the Impact time-step for: (**A**) a 10 m tsunami wave (T=1800 s) and (**B**) a 10 m storm wave (T=10 s). Red dots stand for the position of the boulders.

None of the simulations involving short wind waves reached the position of the blocks, except for sporadic time steps where waves reached the cliff-top, capturing splash processes from their impact on the cliff. Figure 6 illustrates the impact time-steps of a 10 m tsunami wave with a duration of 1800 s and a 10 m storm wave with a period of 10 s, highlighting the differences in wave propagation and impact processes. These results reveal that the formation of Es Bancals boulder deposits is more plausible linked to tsunami waves rather than wind waves.

As aforementioned, most works related to tsunami boulders study formation mechanism of these deposits on slope coast. In addition, most approaches are based on morphometric data and analytic solutions of wave propagation processes. However, wave propagation is a high non-linear process. Complex bathymetries, wave and currents interaction, wave refraction and reflection and positive coupling should be addressed with numerical models that are able to capture these processes. In this sense, to discern whether storm waves or tsunami waves are the most plausible mechanism for the formation of such deposits, requires a transdisciplinary approach, such as the one employed in this study, to enhance our understanding of these processes.

## Conclusions

This study explores the mechanisms behind the formation of cliff-top boulder deposits by combining detailed geomorphometric measurements with numerical modeling, focusing on boulder deposits along the southern coast of Mallorca Island (Balearic Islands, Western Mediterranean).

Numerical results provide strong evidence that tsunami waves are the primary mechanism responsible for the transport and deposition of cliff-top deposits at the study area. In addition, the most probable tsunami directions responsible for these deposits align with seismic sources found in the southern Western Mediterranean (^17,27,32,33^, among others).

The combined analysis of morphometric data and numerical simulations of tsunami wave propagation at impact points, considering wave height and velocity fields, reveals that impact velocities are the key parameter in determining whether boulders can be transported by the flow and the type of mobilization they undergo. Although analytical solutions for wave propagation can approximate wave height and velocity at impact points^19,43^, they do not account for critical processes such as wave interaction with complex bathymetries, propagation, refraction, and other two-dimensional effects. These processes are essential for capturing lateral variations within a given area.

The current approach prioritizes tsunami wave propagation and transformation over generation, highlighting the importance of high-resolution simulations in capturing key wave shoaling and dissipation effects. By simulating tsunami waves that have already traveled from their source, the study effectively represents critical processes such as drawback flow, wave amplification, impact, and flooding–essential for understanding tsunami behavior in shallow coastal areas.

Furthermore, the numerical results align with previous research on tsunami impacts in cliffed environments, reinforcing our understanding of wave amplification and boulder transport in steep coastal settings. However, such studies remain limited, underscoring the need for further research. A comprehensive review of similar deposits in high cliffs is necessary, as some may have been misclassified as wave-driven rather than tsunami-driven, or vice versa.

The high-resolution numerical modeling approach used in this study is particularly relevant for tsunami risk assessment, as it captures key wave transformation processes, including shoaling, dissipation, and impact dynamics in both sloped and cliffed coastal environments. By accurately simulating tsunami wave propagation and its interaction with complex coastal topographies, this methodology provides valuable insights into potential inundation zones, wave amplification effects, and boulder transport mechanisms. Such detailed analyses enhance hazard mapping and coastal planning efforts, enabling more precise risk evaluations and the development of targeted mitigation strategies to protect vulnerable coastal communities from future tsunami events.

## Methods

### Morphometric Analysis and mobilization thresholds

Morphometric analysis has been conducted by identifying the largest boulders along the platform at the study site. For each boulder, the maximum axis (a), the minimum axis (b), and the block thickness (c), representing the largest horizontal dimension, the largest vertical dimension, and the width of the boulder, respectively, have been measured, as shown in Figure 1C, together with their orientation $\rho _{bl}$, bed-slope angle $\theta _{sl}$, height above sea level, and distance from the cliff edge. Orientation has been determined by measuring the azimuth of the long axis relative to the true north. The morphometric values of each boulder have been calibrated using the triangulation method^49^. Morphometric data for both sectors is detailed in Tables 1 and 2.

**Table 1:** Morphometric data of western sector Boulders at Es Bancals.

Boulder number	a (m)	b (m)	c (m)	Direction $\rho _{bl}$ (deg.)	$\theta _{sl}$ (rad.)	Height (m)	Distance to the sea (m)	Boulder volume ($m^{3}$)
1	1.10	0.70	0.35	0.00	0.00	36.30	27.20	0.27
2	1.60	1.37	0.45	227.00	0.36	36.50	28.00	0.99
3	1.07	0.40	0.35	0.00	0.00	37.50	39.00	0.15
4	0.62	0.59	0.32	202.00	1.41	37.20	39.30	0.12
5	1.17	0.52	0.46	0.00	0.00	37.80	45.40	0.28
6	0.80	0.52	0.21	241.00	0.56	37.50	44.20	0.09
7	1.60	0.80	0.25	148.00	0.42	37.70	47.40	0.32
8a	0.72	0.68	0.21	286.00	0.73	35.20	34.80	0.10
8b	1.23	0.53	0.37	239.00	0.63	35.60	36.30	0.24
8c	0.67	0.56	0.15	224.00	0.35	35.80	37.10	0.06
9	1.30	1.00	0.65	199.00	0.21	34.00	29.80	0.85
Average	1.08	0.70	0.34	220.75	0.58	36.46	37.14	0.31
Std.	0.35	0.28	0.14	39.94	0.37	1.22	6.88	0.31

**Table 2:** Morphometric data of eastern sector boulders at Es Bancals.

Boulder number	a (m)	b (m)	c (m)	Direction $\rho _{bl}$ (deg.)	$\theta _{sl}$ (rad.)	Height (m)	Distance to the sea (m)	Boulder volume ($m^{3}$)
11	2.00	1.05	0.65	0.0	0.00	24.50	23.10	1.36
12	1.30	1.20	1.10	0.00	0.00	24.80	23.90	1.72
13	1.03	0.75	0.56	0.00	0.00	24.80	23.40	0.43
14	1.20	0.80	0.43	246.00	0.54	25.30	26.20	0.41
15	2.00	1.20	0.24	248.00	0.38	24.80	26.90	0.58
16	2.30	1.80	0.30	0.00	0.00	25.70	34.80	1.24
17	2.10	1.20	0.40	233.00	0.21	25.90	38.80	1.01
18	1.80	1.20	0.20	249.00	0.26	26.20	43.60	0.43
19	1.90	0.80	0.50	0.00	0.00	23.70	61.20	0.76
20	0.50	1.20	0.70	0.00	0.00	19.40	51.90	0.42
21	1.80	0.80	0.40	243.00	0.68	17.60	38.30	0.58
22	1.80	1.10	0.80	230.00	0.37	19.10	52.80	1.58
23	1.30	0.70	0.30	234.00	0.65	20.50	62.60	0.27
24	1.30	1.10	0.80	0.00	0.00	18.40	65.00	1.14
25	1.40	0.80	0.70	0.00	0.00	20.30	70.40	0.78
26	1.90	1.90	0.30	0.00	0.00	18.40	65.80	1.08
27	2.40	1.60	0.80	200.00	0.28	18.70	68.20	3.07
28	3.00	2.20	0.80	0.00	0.00	17.00	61.50	5.28
29	2.00	1.70	0.60	189.00	0.52	18.80	69.80	2.04
30	1.80	1.60	0.80	230.00	0.17	18.30	72.60	2.30
31	3.10	1.70	0.60	235.00	0.68	19.10	73.90	3.16
32	3.20	2.40	0.50	243.00	0.52	15.30	54.70	3.84
33	3.40	2.70	0.60	0.00	0.00	14.90	51.70	5.51
34	2.70	1.90	0.40	219.00	0.35	16.20	63.00	2.05
35	2.10	1.30	0.30	180.00	1.01	18.60	42.30	0.82
36	1.30	0.90	0.40	204.00	0.93	18.60	43.40	0.47
37	3.10	1.40	0.40	210.00	0.26	18.50	43.00	1.74
38	4.00	2.10	0.80	218.00	0.21	17.40	35.90	6.72
39	1.20	1.00	0.20	194.00	0.35	18.10	40.40	0.24
40	1.10	0.70	0.30	185.00	0.56	18.30	43.70	0.23
Average	2.00	1.36	0.53	220.53	0.47	20.24	49.09	1.71
Std.	0.82	0.54	0.23	22.81	0.24	3.45	16.11	1.69

Mobilization thresholds considering the subaerial, submerged and joint bounded block (JBB) conditions in terms of wave height and velocity have been computed by considering the modified^12^ equations^13,14^ as summarized in Table 3.

**Table 3:** Mobilization thresholds equations for subaerial, submerged and joint bounded block conditions, where $\rho _b$ and $\rho _w$ stand for boulder and water density and set to 2.6 and 1.025 g cm$^{-3}$, respectively, V is the corrected boulder volume, $\mu$ is the static friction coefficient (set to 0.65), $\theta _{sl}$ is the bed slope angle of the pre-transport setting, $C_d$ is the drag coefficient (set to 1.95,^18^), $C_l$ is the lift coefficient (set to 0.178,^18^), *q* is the empirical aspect ratio of each boulder set to 0.73^18^ and g is the acceleration due to gravity.

	Subaerial	Submerged	Joint bounded block
Tsunami wave height $H_t$ (m)	$H_t = 0.5 \mu \dfrac{\rho _b V C_d a c q}{\rho _w}$	$H_t = 0.25 \left( \dfrac{\rho _b - \rho _w}{\rho _w}\right) ^2 a C_d \left( \dfrac{ac}{b^2}\right) + C_l$	$H_t = \dfrac{(\rho _b - \rho _w) V (\cos \theta _{sl} + \mu \sin \theta _{sl})^2}{\rho _w C_l a b q}$
Wind waves height $H_s$ (m)	$H_s = 2 \mu \dfrac{\rho _b V C_d a c q}{\rho _w}$	$H_s = \left( \dfrac{\rho _b - \rho _w}{\rho _w}\right) ^2 a C_d \left( \dfrac{ac}{b^2}\right) + C_l$	$H_s = \dfrac{(\rho _b - \rho _w) V (\cos \theta _{sl} + \mu \sin \theta _{sl})^{0.5}}{\rho _w C_l a b q}$
Velocity thresholds *u* (m/s)	$\text {Sliding: } u^2 = 2 \dfrac{(\rho _b/\rho _w - 1) g c (\cos \theta _{sl} + \mu _s \sin \theta _{sl})}{C_d (c/b) + \mu _s C_l}$		
	$$\text {Rolling: } u^2 = 2 \dfrac{(\rho _b/\rho _w - 1) g c (\cos \theta _{sl} + \mu _s \sin \theta _{sl})}{C_d (c^2/b^2) + \mu _s C_l}$$		
	$$\text {Saltation: } u^2 = 2 \dfrac{(\rho _b/\rho _w - 1) g c \cos \theta _{sl}}{C_l}$$		
	$$\text {Saltation/lifting: } u^2 = 2 \dfrac{(\rho _b/\rho _w - 1) g c (\cos \theta _{sl} + \mu _s \sin \theta _{sl})}{C_l}$$		

### Numerical modeling

The FUNWAVE-TVD (Fully Nonlinear Boussinesq Wave model with Total Variation Diminishing scheme), developed by^30^, is a numerical model designed for the comprehensive simulation of fully nonlinear and dispersive water wave phenomena. The model accurately represents a range of wave conditions, including interactions with complex coastal bathymetry, shoaling, and breaking. Widely utilized in coastal engineering and research, FUNWAVE-TVD is particularly effective in studying nearshore processes, providing insights into coastal morphodynamics, sediment transport, and wave-induced changes in coastal areas. Notably, its utilization extends to tsunami research, where its robust capabilities contribute to understanding and modeling the complexities of tsunami wave propagation and inundation dynamics. As developed in^50^, the computational domain embraces 6075x6000 m with an equispaced grid of 10x10 m. Topobathymetry has been generated by considering the high-resolution 2.5x2.5m LIDAR digital terrain model (DTM) topography^51^ and the bathymetry from the Balearic Islands Territorial Information System.

Since we focus on the high-resolution solution of the tsunami waves propagation towards the coast, accounting for shallow water conditions and physical processes (i.e. dissipation, shoaling, refraction, diffraction, reflection and wave breaking), the model is forced at the off-shore boundary of the computational domain (40 m depth), following preliminary results shown in^50^, considering different off-shore wave conditions. A total of 360 simulations were conducted, covering off-shore wave heights $H_0$ from 5 to 20 m, periods *T* from 300 to 1800 s, and directions $\theta _0$ ranging from $180^{\circ }$ (S) to $225^{\circ }$ (SW), matching the direction of potential tsunamis forcing origin^32,36^, an assuming that the MSL is the current reference position. Each simulation represents a single offshore wave condition defined by the tuple [$H_0$, *T*, $\theta _0$] imposed at the wavemaker position. Periodic lateral boundary conditions are considered. Note that, to avoid numerical artefacts or instabilities in the results, the lateral extent of the computational domain is sufficiently long on both sides of the area of interest.

**Table 4:** Mobilization velocity thresholds for western sector boulders at Es Bancals.

Boulder number	Subaerial		Submerged		Subaerial/submerged			JBB		
	Ht (m)	Hs (m)	Ht (m)	Hs (m)	Sliding *u* (m/s)	Rolling *u* (m/s)	Saltation *u* (m/s)	Ht (m)	Hs (m)	Salt./lift *u* (m/s)
1	0.41	1.62	0.49	1.98	3.11	4.18	7.70	2.07	8.28	7.70
2	0.79	3.17	1.33	5.31	4.57	6.97	8.44	3.10	12.42	9.43
3	0.23	0.93	0.17	0.69	2.41	2.56	7.70	2.07	8.28	7.70
4	0.34	1.37	0.37	1.48	2.56	3.34	2.91	1.51	6.04	6.58
5	0.30	1.20	0.22	0.89	2.74	2.91	8.83	2.72	10.88	8.83
6	0.30	1.20	0.44	1.77	2.89	4.17	5.49	1.48	5.92	6.51
7	0.46	1.85	0.88	3.52	3.50	5.39	6.22	1.74	6.96	7.06
8a	0.39	1.58	0.68	2.71	3.22	4.97	5.14	1.46	5.85	6.47
8b	0.31	1.23	0.28	1.13	3.00	3.53	7.12	2.61	10.42	8.64
8c	0.32	1.30	0.64	2.56	2.87	4.53	4.89	1.03	4.12	5.43
9	0.58	2.32	0.55	2.19	3.97	4.82	10.38	4.28	17.11	11.07
Average	0.40	1.62	0.55	2.20	3.17	4.31	6.80	2.19	8.75	7.77
Std.	0.16	0.64	0.33	1.33	0.64	1.25	2.12	0.93	3.74	1.62

**Table 5:** Mobilization velocity thresholds for eastern sector boulders at Es Bancals.

Boulder number	Subaerial		Submerged		Subaerial/submerged			JBB		
	Ht (m)	Hs (m)	Ht (m)	Hs (m)	Sliding *u* (m/s)	Rolling *u* (m/s)	Saltation *u* (m/s)	Ht (m)	Hs (m)	Salt./lift *u* (m/s)
11	0.61	2.43	0.62	2.48	3.85	4.77	10.49	3.84	15.37	10.49
12	0.69	2.78	0.47	1.89	4.17	4.35	13.65	6.50	26.02	13.65
13	0.43	1.74	0.36	1.45	3.28	3.75	9.74	3.31	13.24	9.74
14	0.46	1.85	0.53	2.11	3.64	4.77	7.90	3.03	12.12	9.32
15	0.69	2.78	1.86	7.42	4.09	6.61	6.14	1.66	6.64	6.90
16	1.04	4.17	2.98	11.91	4.53	7.30	7.13	1.77	7.10	7.13
17	0.69	2.78	1.23	4.91	4.19	6.36	8.14	2.63	10.53	8.68
18	0.69	2.78	2.08	8.31	3.94	6.34	5.72	1.34	5.36	6.20
19	0.46	1.85	0.48	1.90	3.36	4.14	9.20	2.96	11.83	9.20
20	0.69	2.78	0.59	2.36	4.10	5.20	10.89	4.14	16.56	10.89
21	0.46	1.85	0.58	2.33	3.62	4.87	7.26	2.81	11.22	8.96
22	0.64	2.55	0.55	2.21	4.28	4.95	11.25	5.52	22.07	12.57
23	0.41	1.62	0.58	2.31	3.36	4.77	6.37	2.11	8.44	7.78
24	0.64	2.55	0.54	2.15	3.97	4.59	11.64	4.73	18.92	11.64
25	0.46	1.85	0.34	1.36	3.40	3.62	10.89	4.14	16.56	10.89
26	1.10	4.40	3.00	12.02	4.62	7.42	7.13	1.77	7.10	7.13
27	0.93	3.71	1.12	4.50	5.02	6.75	11.41	5.39	21.58	12.43
28	1.27	5.10	2.01	8.05	5.41	8.04	11.64	4.73	18.92	11.64
29	0.98	3.94	1.56	6.22	5.18	7.75	9.38	4.23	16.90	11.00
30	0.93	3.71	1.08	4.34	4.93	6.62	11.55	5.19	20.77	12.20
31	0.98	3.94	1.66	6.65	5.17	7.74	8.89	4.21	16.83	10.98
32	1.39	5.56	3.42	13.66	5.86	9.47	8.56	3.52	14.08	10.04
33	1.56	6.25	3.61	14.44	5.74	9.24	10.08	3.55	14.19	10.08
34	1.10	4.40	2.72	10.90	5.16	8.33	7.98	2.75	10.99	8.87
35	0.75	3.01	1.78	7.13	4.16	6.67	5.19	1.92	7.67	7.41
36	0.52	2.08	0.70	2.79	3.71	5.19	6.39	2.65	10.60	8.71
37	0.81	3.24	1.69	6.75	4.51	7.05	8.09	2.68	10.73	8.77
38	1.22	4.86	1.93	7.72	5.59	8.21	11.51	5.27	21.06	12.28
39	0.58	2.32	1.43	5.71	3.72	6.01	5.64	1.37	5.50	6.27
40	0.41	1.62	0.57	2.27	3.37	4.77	6.56	2.12	8.46	7.78
Average	0.79	3.15	1.87	7.49	4.33	6.19	8.88	3.39	13.58	9.40
Std.	0.31	1.25	1.01	4.02	0.77	1.63	2.26	1.39	5.56	1.92

## Data Availability

Data related to boulders position and size is available at Gelabert Ferrer, B., & Roig-Munar, F. X. (2025). Morphometric data of Tsunami Boulder Deposits from Els Bancals (Mallorca, NW Mediterranean) [Data set]. Zenodo. doi.org/10.5281/zenodo.16901281.
